# Rumen-Derived Consortia Shaped by Substrate-Specific Enrichment Show Specialized Lignocellulose Utilization, Diversified Hydrogen Metabolism, and Cryopreservation Stability

**DOI:** 10.3390/microorganisms14051149

**Published:** 2026-05-19

**Authors:** Ajay Badhan, Chunli Li, Le Luo Guan, Tim A. McAllister

**Affiliations:** 1 Agriculture and Agri-Food Canada, Lethbridge Research and Development Centre, Lethbridge, AB T1J 4B1, Canada; 2 Faculty of Land and Food Systems, University of British Columbia, 2205 East Mall, Vancouver, BC V6T 1Z4, Canada

**Keywords:** rumen microbiota, enrichment culture, probiotics, diversity, volatile fatty acid, cryopreservation, microbial consortia

## Abstract

Efficient utilization of lignocellulosic biomass by the rumen microbiome is critical for improving feed efficiency in ruminants, yet the development of stable, functionally specialized microbial consortia remains limited. This study aimed to assemble substrate-adapted rumen microbial consortia using an ecology-guided enrichment approach. Rumen fluid collected from cannulated Angus × Hereford heifers was sequentially enriched over 10 generations on four substrates with distinct cell wall characteristics: alfalfa, barley straw, carboxymethyl cellulose (CMC), and xylan. Fermentation parameters, including gas production and volatile fatty acids (VFAs), and bacterial community dynamics were analyzed, and selected consortia (alfalfa and xylan) were evaluated for stability following one month of cryopreservation. Across enrichments, total VFA concentrations declined (e.g., xylan: 109.8 mM (G0) to 56.37 mM (G10)), accompanied by reduced gas production and decreased alpha diversity, indicating substrate-driven selection. Distinct functional profiles emerged, including increased propionate in alfalfa consortia, higher acetate in barley straw, lactate–propionate cross-feeding with CMC, and caproate production (6.3 mM at G10) in xylan enrichments associated with *Caproiciproducens* and *Megasphaera*. Cryopreserved consortia retained core community structure and fermentation characteristics upon revival. These results demonstrate that substrate-driven enrichment can generate stable, functionally specialized rumen consortia and provide a framework for developing ecologically compatible microbial communities with potential applications in improving rumen fermentation efficiency.

## 1. Introduction

Global demand for meat and milk is projected to increase substantially by 2050 due to population growth, rising incomes, and urbanization [1]. Ruminant livestock are central to meeting this demand because of their unique ability to convert lignocellulosic biomass into high-quality animal protein, contributing approximately 45% of global dietary protein intake [2]. However, the efficiency of ruminant production systems remains relatively low, limiting profitability and increasing environmental burdens [2]. Enteric methane (CH_4_) emissions represent a loss of 4–12% of dietary energy and are a major contributor to agricultural greenhouse gas emissions [3]. Improving rumen fermentation efficiency is therefore a key objective, as it offers simultaneous benefits for animal productivity and environmental sustainability.

The rumen microbiome is a critical determinant of feed efficiency and methane emissions, with both community composition and metabolic activity influencing fermentation outcomes [4,5]. Efficient rumen systems are typically characterized by functionally specialized microbial communities that exhibit optimized electron flow, reduced metabolic redundancy, and tight coupling between substrate degradation and hydrogen utilization pathways [6,7,8]. Consequently, manipulation of the rumen microbiota has been proposed as a strategy to enhance fermentation efficiency and reduce environmental impacts.

Probiotic approaches have been explored to modulate the rumen microbiome; however, their effectiveness has been inconsistent [9,10,11]. A major limitation is the poor colonization and persistence of introduced strains, particularly when non-rumen-derived microorganisms are used, as they are often unable to compete with indigenous microbiota or adapt to rumen conditions such as strict anaerobiosis, low redox potential, and dynamic physicochemical environments [12,13,14,15,16]. In contrast, rumen-derived microbial consortia may offer improved ecological compatibility and functional integration [15,16,17]. Multi-species consortia are also expected to provide enhanced stability through complementary metabolic functions and cross-feeding interactions [17]. Therefore, developing rumen-derived probiotic consortia that can efficiently target specific, recalcitrant feed fractions while partitioning hydrogen away from methane toward beneficial volatile fatty acids (VFAs) may enhance feed efficiency.

Two contrasting approaches exist for constructing such consortia. Rational design based on axenic strain assembly aims to combine well-characterized isolates with defined metabolic roles. However, this approach remains limited in the rumen context due to incomplete knowledge of the metabolic capacities and efficiencies of many uncultured microorganisms and insufficient understanding of their interaction networks, including cross-feeding and syntrophic dependencies. Alternatively, ecology-guided enrichment approaches use selective pressures to allow microbial communities to self-organize into functionally coherent systems. While enrichment strategies are widely used in anaerobic systems, their application for generating stable, substrate-adapted rumen consortia and their functional implications remain underexplored. There is currently limited ability to construct stable, functionally coherent rumen consortia with predictable metabolic outputs.

Substrate composition is a key ecological driver of microbial community assembly, as different lignocellulosic fractions impose distinct metabolic and thermodynamic constraints. For example, soluble substrates may favor rapid fermenters and propionate production, whereas recalcitrant structural components select for fibrolytic organisms and alternative hydrogen sinks. However, it remains unclear whether substrate-driven enrichment can reproducibly generate stable microbial consortia with predictable metabolic outputs and resilience to perturbations such as storage.

Based on these considerations, we hypothesized that (i) sequential enrichment on structurally distinct substrates would drive deterministic selection of substrate-specific microbial consortia, (ii) these consortia would exhibit distinct fermentation profiles reflecting substrate composition and associated metabolic networks, and (iii) the resulting communities would retain structural and functional stability following cryopreservation. To test these hypotheses, rumen fluid was enriched over 10 generations on four structurally distinct substrates—alfalfa (dicot, nutrient and protein-rich as compared to other substrate), barley straw (BS, monocot, low-quality fiber with high NDF, ADF and lignin content as compared to other substrates), carboxymethyl cellulose (highly ordered via intracrystalline hydrogen bonds conferring strong recalcitrance to ADF), and acetylated xylan (comprising partially acetylated β-1,4-linked xylose backbones with ferulic acid-crosslinked arabinoxylan side chains imparting recalcitrance to NDF). Fermentation characteristics and microbial community dynamics were assessed, and selected consortia were evaluated for post-cryopreservation recovery. Overall, this study demonstrates that substrate-driven ecological selection can generate specialized, stable rumen microbial consortia, providing a framework for developing ecologically compatible microbial systems with potential applications in improving rumen fermentation efficiency and related biotechnological processes.

## 2. Materials and Methods

### 2.1. Chemicals

Sodium bicarbonate (NaHCO_3_; Sigma-Aldric, Oakville, ON, Canada, catalogue no. S5761), potassium phosphate dibasic (K_2_HPO_4_; Sigma-Aldric, catalogue no. S3264), potassium phosphate monobasic (KH_2_PO_4_; Sigma-Aldrich, catalogue no. P5655), ammonium sulfate ((NH_4_)_2_SO_4_; Fisher, Pittsburgh, PA, USA catalogue no. A702), sodium chloride (NaCl; Sigma-Aldrich, catalogue no. S9625), Magnesium sulfate heptahydrate (MgSO_4_·7H_2_O; Sigma-Aldrich, catalogue no. M1880), calcium chloride dihydrate (CaCl_2_H_4_O_2_, or CaCl_2_·2H_2_O; Sigma-Aldrich, catalogue no. C3881), L-cysteine hydrochloride (Sigma-Aldrich, Oakville, ON, Canada, catalogue no. C1276), yeast extract (OXOID REF LP0021), bacto casitone (BD REF 225930), vitamin supplement (ATCC, VA, USA, catalogue no. MD-VS), sodium 2-mercaptoethane sulfonate (C2H_5_NaO_3_S_2_, Sigma-Aldrich, catalogue no. M1511), resazuarin sodium salt (Sigma-Aldrich, catalogue no. R7017), carboxymethyl cellulose (Sigma-Aldrich, catalogue no. C5013), arabinoxylan (Wheat Flour; Low Viscosity, Neogen, Lexington, KY- P-WAXYL), xylan (Birchwood, partially acetylated, Neogen-PACXYL), RNA-later (ThermoFisher, ON, Canada, Catalogue no. AM7021), tri-zol reagent (ThermoFisher, Catalogue no. 15596026).

### 2.2. Experimental Design for Enrichment Culture Experiments

Enrichment in vitro batch cultures were set up in 3 replicate serum vials. Barley straw (BS), alfalfa, carboxymethyl cellulose (CMC) and xylan (mix of arabinoxylan (50%) and acetylated-xylan (50%) were weighed (0.3 g) into (125 mL) serum vials. Rumen fluid from 4 different sites within the rumen was collected from four cannulated Angus × Hereford heifers fed a barley straw (15%), corn silage (82%) and standard feedlot supplement (3%). Collected rumen fluid was strained through four layers of cheesecloth and equal volumes from each cow were combined and anaerobically maintained under a stream of O_2_-free CO_2_. The inoculum was prepared by mixing rumen fluid and MC medium [18] with the ratio of 1:4. Inoculum (30 mL) was transferred to each vial under a stream of O_2_-free CO_2_. Vials were sealed with rubber stoppers and placed in a rotary shaker (125 rpm) in an incubator at 39 °C for 72 h. This was the initial batch of enrichment cultures, referred to as “G0”. Gas pressure in each vial was measured after 24, 48 and 72 h of incubation by inserting a 23-gauge (0.6 mm) needle attached to a pressure transducer (model PX4200-015GI, Omega Engineering, Inc., Laval, QC, Canada). After completion of 72 h of incubation, a 6 mL sub-sample was collected and inoculated (injected using syringe, pre-washed with 1 g L^−1^ of L-cysteine hydrochloride) from G0 to G1 sealed vials (50 mL) containing fresh MC media (24 mL) with respective substrate (0.3 g), under CO_2_. This sub-culturing step from the previous generation to the subsequent generation was repeated after 72 h of incubation up to G10. At the same time, the previous batch was sampled for VFA analysis (1.5 mL added to 300 µL of 25% metaphosphoric acid) by gas chromatography [19]). The remaining cultures were centrifuged at 12,000× *g* for 20 min at 1 °C to collect supernatant and pellets from G0, G3, G6 and G10. RNAlater (ThermoFisher, Catalogue no. AM702; 1.5 mL) was added to biomass pellets before storage at −80 °C.

Following completion of the 10th generation (G10- after 72 h of incubation) for alfalfa and xylan enrichments, a 6 mL aliquot of the active G10 culture was harvested and mixed in a 1:1 ratio with 6 mL of anaerobic, fresh MC− medium supplemented with glycerol to serve as a cryoprotectant, (final concentration of 15% glycerol (*v*/*v*)). The prepared mixture was immediately transferred into 2-mL sterile, cryogenic vials (Corning, MA, USA, catalogue no. 430488) and flash-frozen in liquid nitrogen and subsequently moved to a −80 °C freezer and stored for one month. To revive the consortia, vials were removed from storage and thawed at room temperature (20 °C) under CO_2_. Once thawed, contents of the vials were inoculated into 24 mL of fresh MC medium containing 0.3 g of the respective substrate (either alfalfa or xylan) and incubated at 39 °C for 72 h, during which the total gas production and samples for VFA profiles were collected as described above.

### 2.3. DNA Extraction

Total DNA from the feed particle attached ruminal microbes was extracted as described previously [20]. Briefly, the biomass pellets obtained from enriched cultures were thawed on ice and centrifuged at 4 °C for 10 min at 12,000× *g*. Pellets were manually ground to a fine powder using a mortar and pestle for 5 min in liquid nitrogen. Ground samples were placed in 2 mL microcentrifuge tubes, and 1.5 mL of TRIzol reagent was added and samples were allowed to stand for 5 min, after which 0.3 mL of chloroform was added and the tubes were shaken vigorously for 15 s. The samples were then allowed to stand at room temperature for 10 min. Subsequently, samples were centrifuged at 12,000× *g* for 15 min at 4 °C. The aqueous phase was removed and DNA was extracted from the remaining interphase and organic phase by precipitation with 100% ethanol. The DNA pellet was washed twice in 0.1 M trisodium citrate, 10% ethanol solution and dried under vacuum, and finally dissolved in 8 mM NaOH.

### 2.4. Sequencing and Data Analysis

16S Sequencing was carried out at Biofactorial (Life Sciences Centre, UBC, BC, Canada) using Illumina NextSeq2000 (Illumina, USA). A dual-indexing, one-step 10 ul PCR reaction (30 cycles) was performed on a LabCyte Access Workstation using Quanta repliQa HiFi ToughMix (QuantBio, QIAGEN Beverly, Inc, MA, USA) with 1 ng input DNA and complete “fusion primers” that include Illumina Nextera adaptors plus indices plus V1/V2 region of the 16S rRNA gene (V1–V2 primer set: Ba27F: AGRGTTTGATCMTGGCTCAG and Ba338R: TGCTGCCTCCCGTAGGAGT). Amplicons were quantified using a picogreen assay (Quant-iT™ PicoGreen™ dsDNA Assay Kit, ThermoFisher) and 2 ng of each product was pooled for subsequent cleanup using a Ampure XP PCR cleanup protocol (Beckman, ON, Canada). The pooled library was quantified using a picogreen assay and loaded onto an Illumina NextSeq P1 600 cycle XLEAP Kit using manufacturer’s recommendations with 25% PhiX.

Forward and reverse reads were quality filtered, through denoising, chimera removal, and inference of amplicon sequence variants (ASVs) in R package DADA2 [bioconductor-dada2 (Version 1.34.0)]. Taxonomy was assigned to ASVs using SILVA (silva_132). The phyloseq package in R (within the Galaxy.eu environment; 4.4.2 with Bioconductor 3.20) was used to integrate the sequence table, taxonomy table, and metadata into a single analysis object. Alpha diversity and beta diversity (PCoA (Principal Coordinate Analysis) on Bray–Curtis distance), was performed with the R package (4.4.2). Alpha diversity was assessed using Shannon and Simpson indices calculated at the ASV level with the estimate richness function in phyloseq. Shannon diversity (H′) was used to quantify both richness and evenness, whereas Simpson diversity emphasized dominance structure. Beta diversity was calculated at the ASV level using Bray–Curtis dissimilarity on relative abundance-transformed data. Prior to distance calculation, count data were normalized to relative abundances using total-sum scaling to account for differences in sequencing depth.

ASVs were amalgamated at the phylum and genus rank using taxonomic glomeration for community composition analysis at the phylum and genus level, respectively. Relative abundances were averaged across biological replicates within each substrate group. Only phyla and genus with ≥1% mean relative abundance in at least one substrate were retained, with the remainder grouped as “Other”. Community composition of RF and G10 enrichment cultures was visualized using pie charts arranged side-by-side. For longitudinal analyses across generations (RF, G0, G3, G6, G10), the 10 most abundant genera (based on total summed abundance across samples) were selected. Relative abundances were averaged per substrate and generation and visualized as stacked bar plots.

Differential abundance at the genus level between generations G0 and G10 was performed for each substrate using the DESeq2 R package (4.4.2). The phyloseq object was converted to a DESeq2 dataset, and counts were modeled using a negative binomial generalized linear model with generation as the main factor. Size factors were estimated to normalize for differences in sequencing depth across samples. Log2 fold changes were computed for G10 relative to G0, and Wald tests were applied to assess statistical significance. *p*-values were adjusted for multiple testing using the Benjamini-Hochberg procedure to control the false discovery rate (FDR). Genera with an adjusted *p*-value < 0.05 and absolute log2 fold change > 1 were considered significantly differentially abundant. Volcano plots were generated for each substrate to visualize differential abundance at the genus level. To improve statistical power and visualization clarity, low-abundant genera were optionally filtered prior to plotting. After core genera determination and filtering, ASVs were collapsed to the genera level. For each substrate, genera were ranked by mean abundance across generations. The top 15 genera per substrate were retained for visualization. Genera appearing consistently at high relative abundance across generations were considered core genera, whereas transient or low-abundant genera were considered non-core. Cryopreserved alfalfa and xylan samples were included to track the persistence of genera prior to enrichment. Abundance values were summed per genus within each generation, and normalized on a 0–1 scale within each substrate to enable comparative visualization as Heatmaps (PrevNorm represents (AbundanceSum/max AbundanceSum)).

### 2.5. Statistical Tests

All statistical analyses were performed in R (v4.4.2). VFAs were analyzed using one-way ANOVA (Analysis of Variance) within each substrate followed by Tukey’s post hoc test, and two-way ANOVA was used to assess the effects of substrate, generation, and their interaction. Results are reported as mean ± SEM (Standard error of the mean), with significance set at *p* < 0.05.

Alpha diversity (Shannon and Simpson indices) was calculated at the ASV level using phyloseq and compared using the Kruskal–Wallis test. Beta diversity was assessed using Bray–Curtis dissimilarity on relative abundance data and analyzed by Permutational Multivariate Analysis of Variance (PERMANOVA vegan, 999 permutations) with substrate and generation as factors (*p* < 0.05).

All analyses were conducted in R (version 4.4.2) using Bioconductor and CRAN packages including Phyloseq, DESeq2, ggplot2, ggrepel, dplyr, purrr, and openxlsx.

## 3. Results

### 3.1. Gas Production Across Enrichment Generations

**Figure 1:**
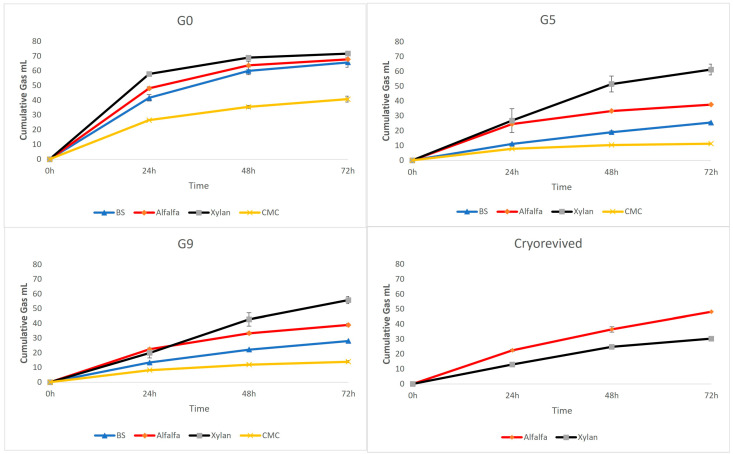
Cumulative gas production (mL) profiles over enrichment generations. Data represent gas volumes measured at 24, 48, and 72 h of incubation for the initial inoculum (G0) through late-stage enrichment (G5 and G9). The panels illustrate fermentation activity across four substrates—barley straw (BS), alfalfa, xylan, and carboxymethyl cellulose (CMC)—as well as the functional recovery of xylan (CP_XL) and alfalfa-based consortia (CP_AF) following one month of cryopreservation at −80 °C.

Across all four substrates—alfalfa, xylan, barley straw (BS), and carboxymethyl cellulose (CMC)—gas production profiles (Figure 1) indicated active fermentation from the initial (G0) through late enrichment generations (G10). In G0, alfalfa and xylan supported the highest cumulative gas production over 24–72 h, whereas BS and CMC consistently yielded lower gas volumes. A similar pattern persisted across G1, G5, and G9, confirming substrate-specific fermentability. A progressive decline in cumulative gas output was observed over successive subculturing. Following cryopreservation, the recovered consortia, produced similar gas on alfalfa while xylan supported lower gas production than G9 after 72 h.

### 3.2. Volatile Fatty Acid (VFA) Profiles Across Generations

**Figure 2:**
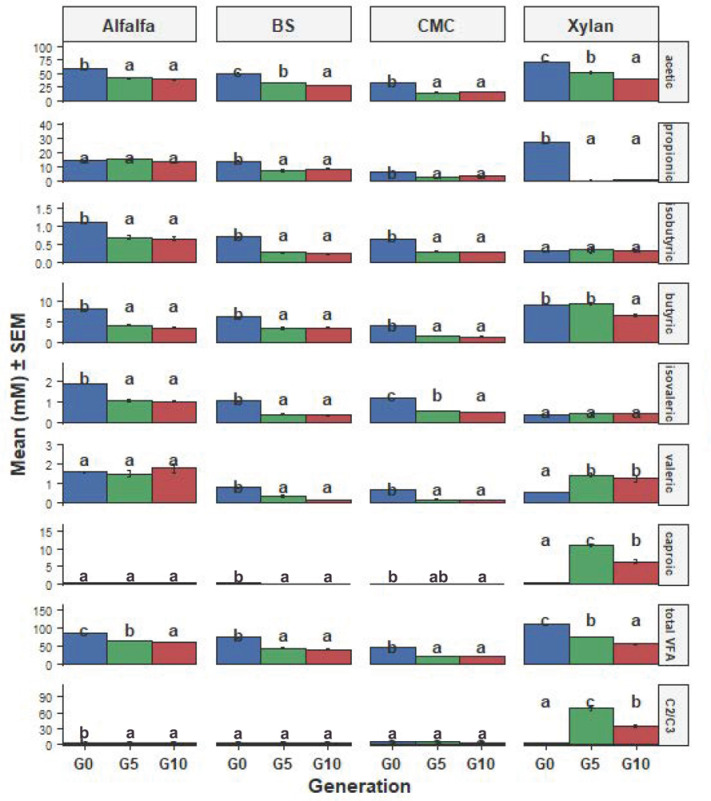
Heat-bar plots showing mean (±SEM) concentrations of individual VFAs, total VFA, and acetate/propionate ratio across microbial generations (G0, G5, G10) for each substrate. Bars are colored by generation, and error bars represent SEM. Statistical differences among generations within each substrate were assessed using one-way ANOVA followed by Sidak-adjusted post hoc tests. Significance is indicated using compact letter displays (CLD), where different letters indicate statistical differences (*p* < 0.05). Generation × Substrate effects were evaluated using two-way ANOVA (Supplementary File S1).

VFA production profiles varied markedly with both substrate type and enrichment generation (Figure 2, Supplementary File S1) and corresponded to observed gas production trends. At G0, xylan yielded the highest total VFA concentration (109.84 mM), dominated by acetic and propionic acids, while CMC resulted in the lowest total VFA (46.06 mM). Alfalfa supported intermediate fermentation (86.50 mM total VFA), with a lower acetate/propionate ratio.

Across generations, total VFA concentrations declined on all substrates, with the sharpest decreases observed for xylan and BS. However, substrate-specific shifts in fermentation patterns emerged. In xylan enrichments, caproic acid (C6) increased from negligible levels at G0 to 6.33 mM at G10, accompanied by a dramatic rise in the acetate/propionate ratio (G0: 2.64 → G5: 69.67 → G10: 34.91), suggesting strong selection for chain-elongating or propionate-limited metabolic networks.

Cryopreserved consortia also exhibited substrate-dependent differences. Xylan-revived cultures generated 67.26 mM total VFA, with a high acetate/propionate ratio (36.90). In contrast, cryopreserved alfalfa (Supplementary File S1) cultures produced lower total VFA (44.99 mM), with a more balanced acetate/propionate ratio (1.86), suggesting restructuring of the consortia during revival.

Volatile fatty acid (VFA) production varied significantly across microbial generations and substrates (Figure 2; Supplementary File S1). Within individual substrates, one-way ANOVA showed clear generational effects for several VFAs, including acetic, propionic, and butyric acids, as well as total VFA concentration and the acetate/propionate ratio (*p* < 0.05) (Supplementary File S1).

Two-way ANOVA further revealed significant Generation × Substrate interactions for most VFAs (Supplementary File S1), indicating that the impact of microbial generation on VFA production depended on substrate type. Consistent with this, pairwise comparisons identified differences between G0 and G5 and/or G0 and G10 in multiple VFA–substrate combinations (*p* < 0.05 to *p* < 0.001, Supplementary File S1). Together, these findings show that shifts in fermentation end-product profiles across generations are strongly substrate dependent.

### 3.3. Diversity Dynamics Across Generations

**Figure 3:**
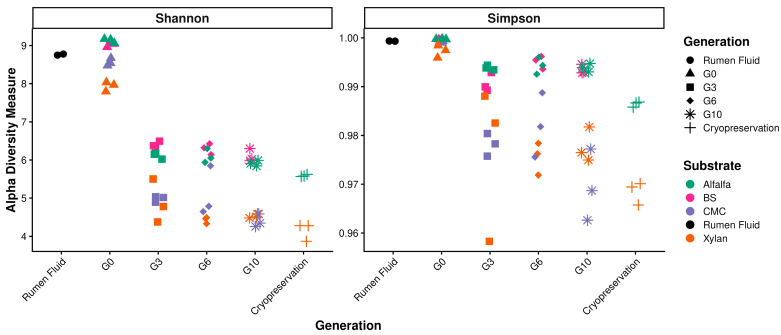
Alpha diversity measures for microbial consortia across enrichment stages. Comparison of Shannon and Simpson diversity indices across sequential generations (G0–G10) and for cryo-revived consortia. Metrics are categorized by substrate type (Alfalfa, BS, CMC, Xylan) and include the baseline rumen fluid (RF) inoculum for comparison.

Alpha diversity metrics (Figure 3) showed a consistent decline from G0 to G10 across all substrates, reflecting strong selective pressures exerted by single-substrate enrichment. The reduction in richness and evenness indicates progressive streamlining of community structure and loss of rare or non-adapted taxa. Simpson diversity remained consistently high across all generations (0.97–1.00), suggesting that community evenness was largely preserved despite the reduction in richness. Cryopreserved samples displayed Shannon and Simpson values comparable to G10, indicating minimal structural disruption following freezing.

**Figure 4:**
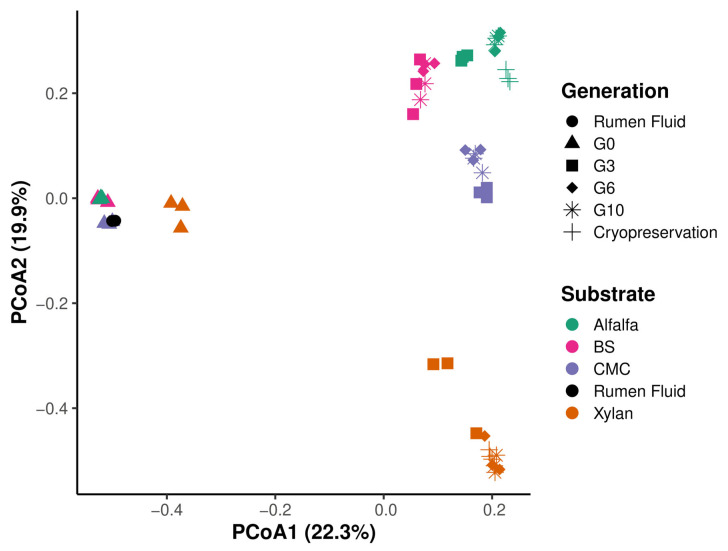
Community clustering via beta-diversity analysis. Principal Coordinate Analysis (PCoA) based on Bray–Curtis distances visualizing the dissimilarity between microbial communities. Data points are color-coded by generation (G0, G3, G6, G10, and Cryopreservation) and shaped by substrate type to show community divergence patterns.

Beta-diversity analysis (Figure 4) demonstrated strong clustering by substrate and enrichment generation. G0 samples from all substrates formed a relatively cohesive community structure reflective of the inoculum, whereas G3–G10 samples diverged. Xylan- and CMC-enriched consortia exhibited the greatest compositional shifts, forming discrete clusters that were distinct from BS and alfalfa communities. Cryopreserved samples clustered near their respective enrichments, confirming preservation of major community members despite minor restructuring. Overall, substrate was the dominant driver of community divergence.

### 3.4. Genus-Level Community Composition

**Figure 5:**
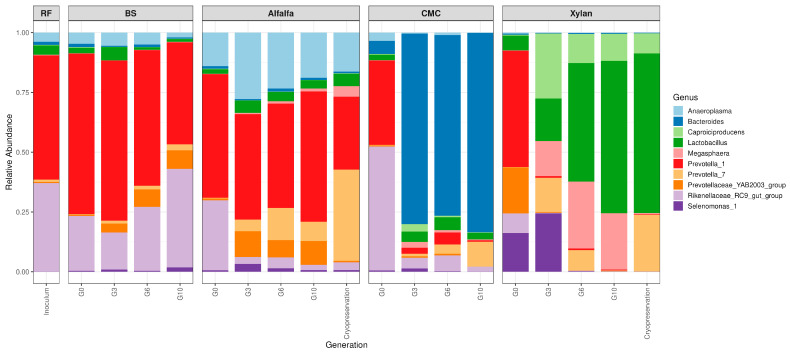
Relative abundance of major (top ten) bacterial genera across substrates and generations. Stacked bar plots depicting the community composition of the top ten most abundant genera identified via 16S rRNA amplicon sequencing. The plots track taxonomic shifts from the initial rumen fluid (RF) inoculum through sequential transfers on specific lignocellulosic substrates.

The inoculum (rumen fluid) exhibited a diverse community dominated by *Prevotella_1*, with substantial contributions from *Rikenellaceae_RC9_gut_group*, and lower abundances of *Anaeroplasma*, *Lactobacillus*, and *Bacteroides*. This baseline composition served as the starting point for all substrate enrichments (Figure 5).

The top ten genus-level profiles reveal clear substrate-driven ecological shifts across generations (G0 → G10), with distinct enrichment patterns for each substrate. The dominant genera identified in the communities included *Anaeroplasma*, *Bacteroides*, *Caproiciproducens*, *Lactobacillus*, *Megasphaera*, *Prevotella_1*, *Prevotella_7*, *Prevotellaceae*_YAB2003_group, *Rikenellaceae*_RC9_gut_group, and *Selenomonas_1* (Figure 5, Supplementary File S2).

Across BS enrichments (G0 → G10), the community became progressively dominated by *Prevotella_1*, which increased sharply and remained the most abundant genus across generations. *Rikenellaceae_RC9_gut_group* remained consistently present and gradually increased as enrichment proceeded. Minor taxa such as *Selenomonas_1*, and *Prevotellaceae_YAB2003_group* also increased across generations (Figure 5).

Alfalfa enrichments supported a more diverse community than BS, but still underwent clear substrate-specific selection. *Prevotella_1*, *Prevotellaceae_YAB2003_group* and *Prevotell_7* remained dominant genera from G3 to G10. *Lactobacillus* was also present across generations. *Anaeroplasma* increased slightly in early (G3) generations, and persisted in subsequent generations indicating adaptation to alfalfa. Minor taxa grown on alfalfa such *Rikenellaceae_RC9_gut_group* declined across generations. Cryopreserved alfalfa communities were also dominated by *Prevotella_1*, *Prevotella_7*, *Megasphaera*, *Lactobacillus* and *Anaeroplasma*, with relative higher abundance for *Prevotella_7* compared to the late-generation community (Figure 5).

CMC enrichments showed strong selective pressure, leading to *Bacteroides* dominating at G3, G6, and G10. Early-generation diversity (G0) including *Prevotella_1*, *Rikenellaceae_RC9_gut_group*, *Selenomonas_1* declined substantially (Figure 5).

Xylan enrichments resulted in a distinct microbial community. *Caproiciproducens*, *Lactobacillus* and *Megasphaera*, increased substantially across generations and became major genera by G6 and G10. *Prevotella_1* markedly decreased as a dominant genus at G0 and was virtually absent by G10. Cryopreserved xylan communities preserved high abundances of *Caproiciproducens* and *Lactobacillus*, and closely matched G10 structure with a drastic reduction in *Megasphaera* (Figure 5). Complete community structure at phylum and genus level is shown in Supplementary Figure S1a,b.

### 3.5. Differential Relative Abundance Between G0 and G10

**Figure 6:**
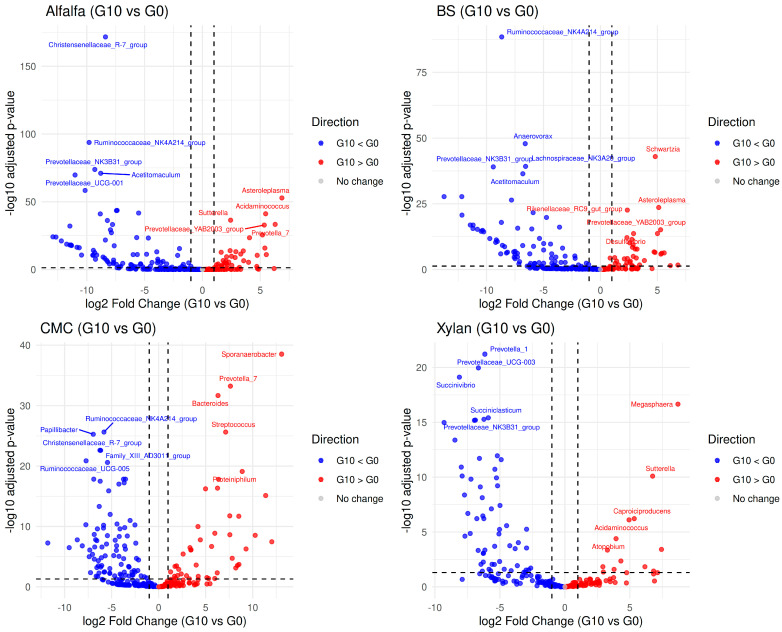
Volcano plots of differential relative abundance between G0 and G10. Comparative analysis of bacterial genera for each substrate, highlighting taxa with a significant change in abundance (adjusted *p*-value < 0.05 and absolute log2 fold change > 1). Red points denote genera with increased relative abundance in late generations, while blue points denote depleted genera. Only top ten genera were named on volcano plot.

Enrichment on Alfalfa resulted in numerous shifts in bacterial community with a strong trend toward depletion (decrease) of several key groups (Figure 6). The most significant decreases were observed for the *Christensenellaceae_R-7_group* and *Ruminococcaceae_NK4A214_group*, (adjusted *p*-value < 0.05 and absolute log2 fold change > 1, Supplementary File S3). Other genera that decreased included *Acetobacterium*, *Prevotellaceae_NK3B31_group*, and *Prevotella_UCG-001*.

In contrast, some genera increased in relative abundance, including *Sutterella*, *Acidaminococcus*, *Asteroleplasma*, *Prevotella_7* and *Prevotellaceae_YAB2003_group* (Figure 6).

*Ruminococcaceae_NK4A214_group* was reduced by repetitive culturing on BS, followed by *Anaerovorax*, *Prevotellaceae_NK3B31_group*, and *Lachnospiraceae_R-3428_group*, whereas genera most significantly enriched on BS included *Schwartzia* and *Asterotplasma*. *Rikenellaceae_RC9_gut_group*, and *Prevotellaceae_YAB2003_group* also showed more moderate increases in G10 compared to G0 (Figure 6).

*Ruminococcaceae* family members were depleted when enriched on CMC, including *Ruminococcaceae_NK4A214_group* and *Ruminococcaceae_UCG-005.* Additionally, *Papillibacter* and *Christensenellaceae_R-7_group* also declined in abundance. Enrichment on CMC promoted the proliferation of *Sporanaerobacter* which exhibited the highest positive fold change with *Bacteroides*, *Streptococcus*, *Proteiniphilum*, and *Prevotella_7* also being enriched (Figure 6).

Enrichment on xylan resulted in decreases in *Prevotella_1*, *Prevotellaceae_UCG-004*. *Succinivibrio*, and *Prevotellaceae_NK3B31_group*. Interestingly, the xylan culture showed a dramatic increase in *Megasphaera* and *Caproiciproducens* (Figure 6).

### 3.6. Dynamics of Core Versus Transient Taxa

The analysis of core versus transient genera across generations (G0 to G10) provided crucial insight into the adaptation and stability of the microbial communities enriched on different substrates. Core taxa were defined by their consistent high abundance presence in all replicates, while transient taxa were those that showed lower or sporadic abundance.

**Figure 7:**
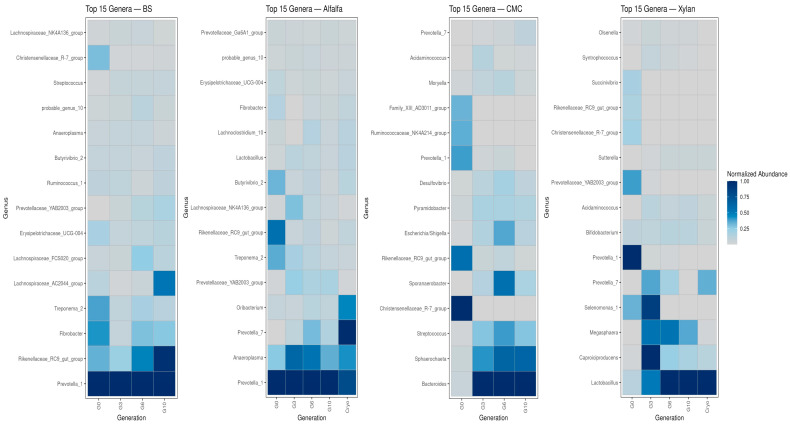
Generational dynamics of enriched genera per substrate. Heatmaps displaying the top 15 enriched genera across enrichment generations (G0–G10) and post-cryopreservation. Color intensity reflects normalized abundance (PrevNorm), calculated within each generation as the summed genus-level abundance divided by the maximum summed abundance observed in that generation.

The microbial consortium enriched with alfalfa exhibited a relatively stable core community across generations. Several taxa were prevalent and stable from G0 through G10. These include *Anaeroplasma*, *Prevotella_1*, *Prevotella_7*, *Prevotellaceae_YAB2003_group*, *Lactobacillus* and *Rikenellaceae_RC9_gut_group*. The stability of these core members suggests they are essential and well-adapted to the in vitro fermentation of alfalfa. A large group of genera remained consistently transient across all generations, including, *Erysipelotrichaeceae*, *Butyrivibrio_2* and *Prevotellaceae_Ga6A1* (Figure 7).

Core taxa with BS enrichment included *Rikenellaceae_RC9_gut_group*, *Prevotellaceae_1*, and *Fibrobacter*. *Christensenellaceae_R-7_group* and *Erysipelatoclostridium*, declined in prevalence and were transient by G6 and G10, suggesting they were either outcompeted or not essential for long-term survival in BS enrichments (Figure 7).

*Bacteroides*, *Sporanaerobacter*, *Streptococcus*, and *Sphaerochaeta* were consistently classified as core genera with CMC from G3 through G10. However, although *Prevotella_1*, *Ruminococcaceae_NK4A214_group*, and *Rikenellaceae_RC9-gut_group* were part of the core community at G0, they became transient later in the process (G6, G10), suggesting a diminished role in the community (Figure 7).

*Prevotella_1* and *Selenomonas_1* showed their strongest signals at early generations on xylan. In contrast, *Megasphaera*, *Caproiciproducens*, *Bifidobacterium* and *Lactobacillus* displayed sustained or increasing normalized abundance through G3–G6 and into G10. The cryopreserved inoculum (“Cryo”) retained several early-stage dominant genera but differed markedly from later enriched communities, confirming that successive subculturing as opposed to cryopreservation shaped the community structure (Figure 7).

## 4. Discussion

### 4.1. Ecology-Guided Enrichment as an Alternative to Model-Driven Design Under Metabolic Uncertainty

Across enrichment generations, reduced gas production, decline in total VFA concentrations, and substrate-specific alterations in VFA profiles (Figure 1 and Figure 2) coincided with decreasing alpha diversity (Figure 3) and shifts in community structure. These results indicated that the substrate was the principal driver of community composition across enrichment generations, while accumulating metabolic byproducts imposed secondary selective pressures that further refined community structure over time (Figure 4, Supplementary File S1). Because all in vitro conditions were identical across treatments, the observed divergence primarily reflects substrate-driven selection modulated by by-product accumulation rather than experimental variability. While this study does not employ a fully model-driven design, such approaches remain constrained in the rumen due to the predominance of uncultured taxa and incomplete knowledge of their metabolic roles. As a result, we utilized an ecology-guided enrichment strategy, allowing substrate-driven selection to organize communities into reproducible and functionally coherent consortia. Initial communities (G0; most reflective of the community in the rumen) were compositionally diverse and included many transient taxa. Repeated transfers selected against these transient taxa and enriched for taxa with known nutrient and fermentation by-product utilization capacities. Hence, at G10 the communities were dominated by a reproducible core taxa e.g., *Prevotella* spp., *Lactobacillus*, *Megasphaera*, (central to succinate/propionate flux in rumen [21]), *Rikenellaceae* RC9_gut_group (functional role includes hemicellulase activity and contribution to the core rumen microbiota [22]) and *Caproiciproducens*, (known to utilize reverse β-oxidation for caproate synthesis and electron disposal [23]) (Figure 5).

### 4.2. Emergence of Metabolic Networks and Cross-Feeding Interactions

A key aspect of this study is the linkage between microbial community structure and fermentation parameters, particularly VFA profiles, which reflect underlying metabolic interactions. The alfalfa enrichments produced the highest total VFA pool and a low acetate:propionate ratio (≈2.9), reflecting rapid soluble-fraction fermentation and expanded succinate→propionate flux, a primary electron sink and nutrient utilization pathway in the rumen [21,24,25]. The short duration in vitro fermentations likely favored microorganisms that grew more rapidly and were more tolerant of higher redox potentials. Under these conditions, bacteria that rapidly utilize soluble carbohydrates, pectin and proteins would have a competitive advantage, particularly given the composition of alfalfa. This may help explain the dominance of *Prevotella*-related taxa and amino acid–utilizing genera, alongside the decline of slower-growing, particle-associated fibrolytic populations. Such temporal selection likely shifted fermentation toward propionate-producers, contributing to the lower acetate/propionate ratio observed. These factors should be considered when interpreting the results relative to in vivo rumen fermentation, where more variable retention times allow greater expression of fibrolytic activity. Barley-straw enrichments showed elevated acetate and a higher A:P ratio (≈3.2 at G10) consistent with intensified fibrolysis of recalcitrant polysaccharides. CMC enrichments evolved toward saccharolytic, lactate-producing networks that quickly engaged lactate-utilizers to form propionate. This syntrophic relationship between lactate producers and utilizers is a well-established stabilization mechanism in rumen fermentation [26]. Several members of the *Veillonellaceae* family showed strong positive fold changes, despite not appearing among the top 10 genera (Supplementary File S3). These taxa are well-known lactate-utilizing, propionate-producing bacteria, indicating that lactate generated by dominant saccharolytic taxa (*Bacteroides*, *Streptococcus* (Supplementary File S3, Supplementary Figure S1)) was redirected toward propionate. Additionally, enrichment of succinate-pathway propionate producers (*Prevotella_7*, *Sporanaerobacter*, *Sphaerochaeta* (Figure 6 and Supplementary File S3)) highlights the role of a distributed cross-feeding network converting soluble sugars and intermediates into propionate. Overall, the results emphasize that low-abundance but metabolically active taxa, acting through both lactate-utilization and succinate-linked pathways, can shape functional outputs in highly selective substrates like CMC. The xylan enrichments developed a chain-elongation phenotype producing caproate (medium-chain carboxylates (MCCAs)). Chain elongation via reverse β-oxidation enables certain rumen bacteria to convert acetate and lactate into medium-chain fatty acids such as caproate [22,27]. These substrate-specific metabolic end-points match the genus-level successions observed in the core/transient analyses, indicating that taxon enrichment that resulted in VFA profile alteration under substrate selection.

### 4.3. Implications for Hydrogen Metabolism

Mechanistically, the metabolic shifts observed across the consortia can be categorized into fermentative -evolving pathways and reductive -consuming (hydrogenotrophic) pathways, the latter representing alternative sinks for reducing equivalents. While the high acetate production in the BS enrichments aligns with typical fermentative evolution, the alfalfa, CMC, and xylan consortia appear to couple generation from fibrolysis to internal sinks like propionate and caproate, potentially reducing the pool of reducing equivalents available to hydrogenotrophic methanogens. While these metabolic shifts are suggested by VFA profiles, it is important to note that direct measurements of hydrogen or methane flux were not performed in our study. Theoretical and experimental studies confirm that redirecting hydrogen to non-methanogenic sinks can limit methanogenesis [24]. The alfalfa consortium’s higher propionate proportion indicates a shift toward electron-consuming pathways that favor host gluconeogenic substrates. Propionate formation is a major gluconeogenic pathway that consumes hydrogen (hydrogenotrophic), directly competing with methanogens for reducing power [24,28]. The xylan consortia provides an additional, possible sink: reverse β-oxidation (chain elongation) by *Caproiciproducens*/*Megasphaera* converts lactate + acetate to butyrate and caproate, consuming electron equivalents in the elongation step. Specific rumen taxa, including *Caproiciproducens*, are known to utilize reverse β-oxidation for caproate synthesis and electron disposal [23,27]. Although caproate concentrations in the xylan-enriched consortia increased from near-undetectable levels at G0 to approximately 11.01 mM at G5, this is below concentrations typically associated with whole-system methanogen inhibition [29,30]. However, this accumulation is sufficient to suggest active reverse β-oxidation metabolism by *Caproiciproducens*. Localized accumulation of caproate in particle-associated microenvironments and continuous redirection of electrons into MCCAs can both (i) reduce hydrogen available to methanogens [23] and (ii) exert local inhibitory pressure on methanogens. Undissociated caproic acid can act as a protonophore, and increasing intracellular levels can destroy the integrity of the cell membrane due to its hydrophobicity [31]. This mechanism is supported by previous reports [29,30,31] that demonstrated a possible inhibitory effect of accumulated medium-chain fatty acids on methanogenic archaea. However, further studies using direct flux measurements are required to confirm these hydrogen redirection pathways.

### 4.4. Stability and Resilience of Enriched Consortia Following Cryopreservation

A notable, practical outcome is the post-cryopreservation revival and retention of core structure. The cryopreserved communities reinstated the same core genera and VFA profiles after recovery. The stability of functional rumen consortia, even after perturbation, is crucial for developing practical inocula [9]. This demonstrates that the consortia are resilient to storage-related perturbation. The cryo-resilience implies that metabolic interactions (cross-feeding, syntrophy) are robust and can be reassembled from preserved inocula. Results presented in this study suggest that alfalfa, barley-straw and xylan consortia are suitable synthetic resource for exploring candidates with potential to improve fiber digestibility, as the increased release of fermentable sugars from lignocellulose and redirection of reducing equivalents into propionate and MCCAs could improve energetic capture per unit feed. These specialized consortia provide a model for exploring how tailored microbial networks might target recalcitrant feed fractions or serve as alternative hydrogen sinks, though their theoretical role in methane mitigation requires confirmation through direct flux measurements.

Effective fiber breakdown and nutrient partitioning are confirmed benefits of optimized rumen communities [5,32,33]. The alfalfa consortium supported soluble-carbohydrate fermentation and propionate production. The *Lactobacillus*-rich cellulolytic CMC consortium shows promise as an enteric probiotic in young ruminants. The compositional and functional characteristics of the CMC-adapted consortium also suggest a practical application during the weaning transition in calves. The dominance of *Lactobacillus* together with *Bacteroides*, *Prevotella_7*, and secondary lactate utilizers such as *Selenomonas* and *Megasphaera* creates a metabolically balanced community capable of converting soluble carbohydrates in starter feeds into lactic, acetic, and propionic acids without an excessive pH decline. These key genera are central to the critical pathway of converting lactate to propionate and acetate in the rumen [26]. The stability of these cross-feeding networks presents a potential avenue for developing consortia that might help stabilize fermentation during weaning transitions. While their ecological compatibility is promising, their ability to prevent metabolic disorders and support animal performance is a hypothesis that requires further testing in live animals. Because these taxa originate from the rumen and maintain cross-feeding relationships that stabilize fermentation and were resilient to storage-related perturbation, the consortium is ecologically compatible with the developing rumen microbiota of weaning calves. The use of *Lactobacillus* as a probiotic for young ruminants has been reported to support gut health [34]. While it is true that translating rumen-derived microbial consortia into a commercial product requires multiple additional steps, the ecological compatibility of these taxa with the developing rumen microbiota provides a strong rationale for their practical potential.

### 4.5. Limitations and Future Perspectives

Despite these advantages, risks such as colonization failure, pH imbalance, or product inhibition remain possible. Risk management strategies for in vivo deployment are informed by both the results presented in this study and established literature. Colonization and persistence risks can be mitigated by (i) leveraging rumen-origin consortia (intrinsic ecological compatibility) as inocula derived from rumen sources show greater persistence and ecological compatibility within the host [35] (ii) co-formulating taxa that occupy complementary niches as redundancy and cross-feeding are expected to enhance persistence. Additional plausible strategies that were not evaluated in the current study and remain topics for future investigation, include (iii) using appropriate delivery/formulations (lyophilisation, encapsulation, bolus or bead systems to lodge consortia on particulate matter) and (iv) co-supplementing with controlled doses of methane inhibitors when necessary, to temporarily reduce methanogen competitiveness and open niches for acetogens or chain-elongators to establish. To avoid pH-related hazards (e.g., lactate accumulation), formulations should include lactate-utilizers (e.g., *Selenomonas*, *Megasphaera*) and be tested across diet types (forage–concentrate ratios) to define safe operating envelopes.

It is important to note that this study employed a substrate-driven enrichment strategy that lacks precise compositional control and relies on inferred metabolic functions. Furthermore, shorter incubation period of in vitro batch culture and soluble substrate used (CMC and Xylan) favour bacterial over fungal or protozoal growth and thus contributions of these eukaryotes were not considered. Future work focused on integrating multi-omics approaches will confirm functional pathways, including hydrogen flux and reverse β-oxidation, and could identify biomarkers of colonization success. Future integration of genome-resolved and controlled co-culture approaches will be necessary to advance toward predictive consortium design.

Beyond rumen applications, these substrate-adapted consortia represent promising platforms for anaerobic digestion and biomass valorization, offering a scalable framework for developing stable and functionally specialized microbial systems.

### 4.6. Conclusions

This study demonstrated that ecology-guided enrichment is a robust strategy for assembling stable, functionally specialized rumen microbial consortia from complex inoculum. By utilizing distinct lignocellulosic substrates, we successfully selected for reproducible core communities capable of specialized metabolic outputs, including enhanced propionate production and the emergence of a caproate-producing chain-elongation phenotype. The demonstrated stability of these consortia following cryopreservation further confirms their potential as resilient, storable inocula. Ultimately, this framework provides a scalable approach for developing ecologically compatible microbial systems with applications spanning animal nutrition (probiotic) and broader biomass valorization.

## Data Availability

The data presented in this study are openly available in NCBI at https://www.ncbi.nlm.nih.gov/bioproject/1433565 (accessed on 17 April 2026), PRJNA1433565.

## Supplementary Materials

The following supporting information can be downloaded at: https://www.mdpi.com/article/10.3390/microorganisms14051149/s1, Supplementary File S1. Statistical analyses and summary data for volatile fatty acid production; Supplementary File S2: Raw phyloseq and relative abundance data at genus and family level; Supplementary File S3: Results from DESeq2 with statistical summary of differential abundance; Supplementary Figure S1: Genus-level community composition of rumen inoculum (RF) and G10 enrichment cultures.
