# Supplementary figures and images for: Rumen-Derived Consortia Shaped by Substrate-Specific Enrichment Show Specialized Lignocellulose Utilization, Diversified Hydrogen Metabolism, and Cryopreservation Stability
Source: Microorganisms. 2026 May 19;14(5):1149. doi: 10.3390/microorganisms14051149 (PMC13209954; doi:10.3390/microorganisms14051149)

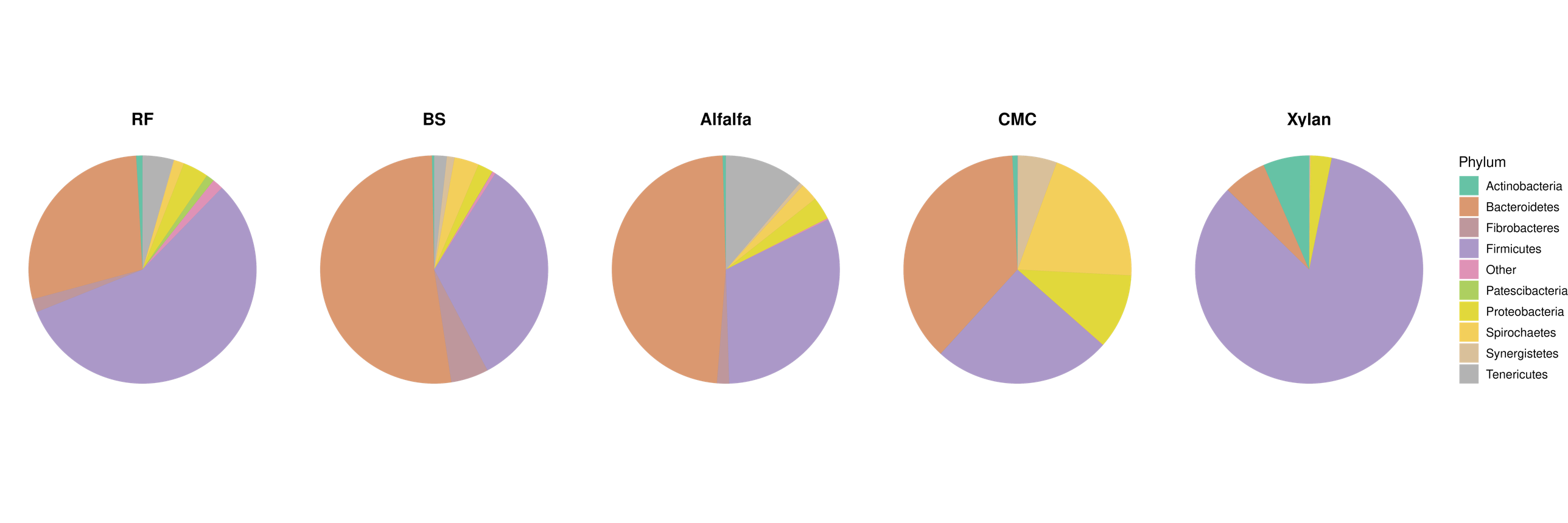

Supplement: Supplementary file 1 [file microorganisms-14-01149-s001.zip › Supplementary Figure S1a,Phylum_RF_vs_G10_1percent.tiff]

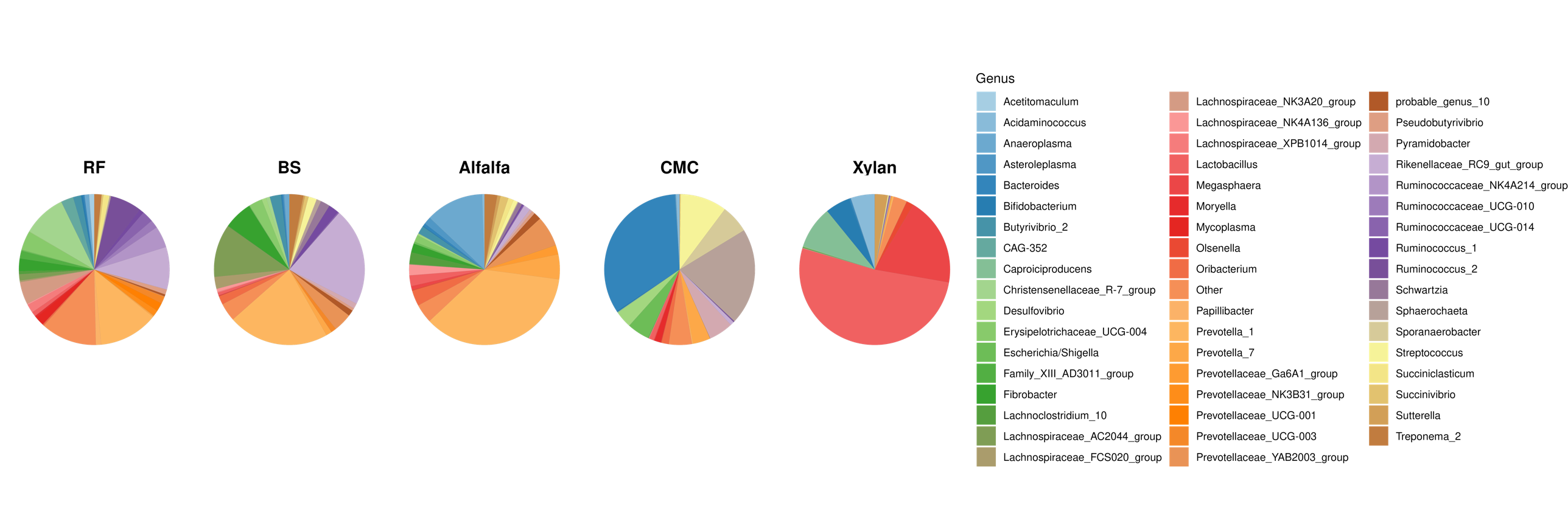

Supplement: Supplementary file 1 [file microorganisms-14-01149-s001.zip › Supplementary Figure S1b,Genus_RF_vs_G10_1percent.tiff]
